# A two-threshold algorithm using donor-derived cell-free DNA fraction and quantity to detect acute rejection after heart transplantation

**DOI:** 10.1016/j.ajt.2025.04.021

**Published:** 2025-05-05

**Authors:** Paul J. Kim, Michael Olympios, Konstantinos Sideris, Eleni Tseliou, Taylor Y. Tran, Spencer Carter, Alison Brann, Navchetan Kaur, Sandra A. Carey, Sangeeta Bhorade, Yen-An Chen, David Barnes, Ebad Ahmed, Jing Xie, Adam Prewett, Matthew Rabinowitz, Bernhard G. Zimmermann, Michelle S. Bloom, Zachary Demko, Eric Adler, Josef Stehlik

**Affiliations:** 1 UC San Diego Health, San Diego, California, USA; 2 Natera, Inc, Austin, Texas, USA; 3 University of Utah School of Medicine, Salt Lake City, Utah, USA

**Keywords:** donor-derived cell-free DNA, heart transplant, acute rejection

## Abstract

Donor-derived cell-free DNA (dd-cfDNA) is a promising biomarker of acute rejection (AR) after heart transplantation (HTx). dd-cfDNA, measured as a fraction of total cfDNA, can be affected by changes in total cfDNA whereas dd-cfDNA quantity can mitigate this impact. This study investigated the performance of a 2-threshold algorithm (2TA) that combines dd-cfDNA fraction (dd-cfDNA%) and donor-quantity score (DQS). A total of 808 plasma samples were prospectively collected for dd-cfDNA testing from 187 adult HTx patients with contemporaneous endomyocardial biopsies. cfDNA was analyzed by a single nucleotide polymorphism-based next-generation sequencing workflow; dd-cfDNA% and DQS were measured using the sequencing reads and single nucleotide polymorphism genotypes. Both dd-cfDNA% and DQS were significantly higher in AR than in non-AR samples (*P* < 10^−14^). Considering samples exceeding either dd-cfDNA% = 0.26% or DQS = 18 copies/mL as positive, the 2TA demonstrated 86.5% sensitivity and 83.6% specificity for AR detection and an area under the curve of 0.881. Compared to dd-cfdNA% alone, performance improved with a mean net reclassification index of 16.4% (standard deviation: 4.0%; *P* =.015) and a 37.3% reduction in the number of the false positive cases compared to the previously established cutoff of 0.15%. Combining dd-cfDNA fraction and quantity estimate in a 2TA may improve AR detection accuracy in HTx recipients compared with dd-cfDNA% alone.

## 1. Introduction

With decreased acute rejection (AR) rates due to modern immunosuppression regimens, the risk-benefit ratio of endomyocardial biopsies (EMBs) for surveillance of acute rejection (AR) is shifting in favor of noninvasive monitoring.^1–9^ One such noninvasive assay is the measurement of donor-derived cell-free DNA (dd-cfDNA) in plasma.^10–14^ dd-cfDNA can be quantified by taking advantage of the genetic differences between an organ donor and recipient. dd-cfDNA levels in the blood increase during allograft rejection, making it a useful biomarker for surveillance of adult and pediatric heart transplantation (HTx) recipients.^15–21^

In a prior study, we described the performance characteristics for a novel, clinically available dd-cfDNA assay for the detection of AR in HTx recipients (the Prospera^1^ Heart test, Natera Inc, Austin, Texas, USA).^19^ Many studies reporting on the testing performance of dd-cfDNA, including our prior work, measured dd-cfDNA as a fraction of total cfDNA (dd-cfDNA%).^18,19^ Total cfDNA consists mostly of recipient-derived cfDNA (rd-cfDNA), with 90% originating from the recipient leukocytes.^22,23^ However, rd-cfDNA levels can be affected by factors unrelated to allograft injury, such as infection,^24^ recipient age,^25^ surgery,^26^ and hemodialysis.^27^ As a result, to avoid false negative results, the recommended cutoffs for dd-cfDNA% have been set relatively conservatively, with the trade-off being more false positive results.

Prior studies in kidney^22,28^ and heart transplant^19,29^ have suggested that measurement of dd-cfDNA quantity could improve testing accuracy.^30,31^ In this study, we tested the hypothesis that combining the dd-cfDNA fraction (dd-cfDNA%) with dd-cfDNA quantity estimate (donor-quantity score; DQS) will improve the performance for detecting AR in HTx patients compared with dd-cfDNA% alone.

## 2. Methods

### 2.1. Study design and sample collection

This analysis is an extension of the DEDUCE study,^19^ an observational study of adult HTx recipients from the University of Utah Medical Center and University of California, San Diego Health undergoing EMB as part of routine care. Eligible subjects included HTx patients who were >18 years of age. Patients who were pregnant, had multiorgan transplants, or a history of other solid organ or stem cell transplants were excluded. Additionally, samples were excluded from analysis of performance if they were drawn during treatment for rejection or if they were drawn <28 days post-HTx.

Plasma samples were collected prospectively, contemporaneously with EMB, from October 2020 to April 2022 and shipped at room temperature to Natera’s Clinical Laboratory Improvement Amendments-certified and College of American Pathologists-accredited laboratory for processing. Sample collection, handling and processing were performed as previously described.^19^ A subset of samples from the DEDUCE study that were frozen immediately following collection was excluded from the present study to align sample storage conditions with clinical practice. Clinical data were extracted from the electronic medical record. The University of Utah Medical Center and the University of California, San Diego Health institutional review boards approved the study (IRB numbers 00094302 and 201821, respectively). This study adheres to the principles of the Declaration of Helsinki and the International Society for Heart and Lung Transplantation Statement on Transplant Ethics.

### 2.2. dd-cfDNA analysis

**Figure 1.**
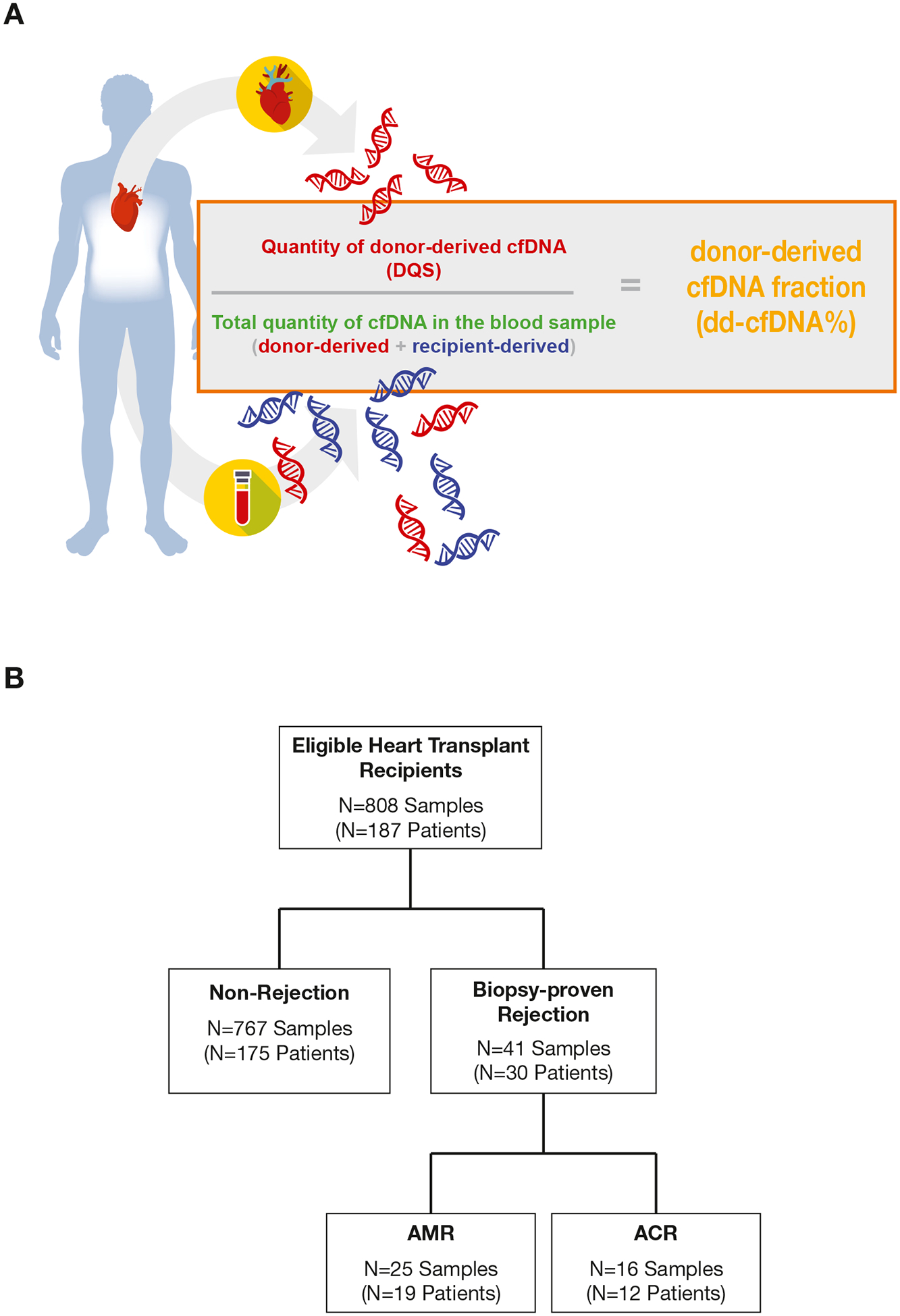
Overview of dd-cfDNA% and DQS and study cohort. (A) dd-cfDNA% represents the proportion of DQS divided by the total quantity of cfDNA. (B) Flow diagram for the study. ACR, acute cellular rejection; AMR, antibody-mediated rejection; dd-cfDNA, donor-derived cell-free DNA; DQS, donor-quantity score.

A massively-multiplexed polymerase chain reaction (PCR) assay (the Prospera test, Natera, Inc) targeting 13,926 single nucleotide polymorphisms was performed. Amplicons were sequenced on the Illumina NextSeq500 with an average of 15 million reads per sample. The analysis of sequencing reads and single nucleotide polymorphism genotyping data enabled the calculation of dd-cfDNA% and estimation of DQS using proprietary algorithms (Fig. 1A).^30,31^ dd-cfDNA% represents the percentage of dd-cfDNA relative to the total cfDNA in plasma. DQS is an estimate of the concentration of dd-cfDNA in plasma reported as genomic copies per milliliter (cp/mL). A grid search approach was used to find the optimal dd-cfDNA% and DQS thresholds for the 2-threshold algorithm (2TA). Using a 2-dimensional grid consisting of incremental DQS values from 8 cp/mL through 35 cp/mL (at increments of 1 cp/mL) and dd-cfDNA% values between 0.1% and 0.5% (at increments of 0.01%), a total of 1,148 threshold combinations were considered. For each combination, patient samples were subsampled 10,000 times, calculating sensitivity, specificity, and area under the curve (AUC) at each iteration, allowing us to construct confidence intervals (CIs) for each metric. Local maxima for the sum of sensitivity and specificity were identified, and the optimal combination that ensured adequate detection of acute cellular rejection (ACR) and/or antibody-mediated rejection (AMR) was selected. As a comparator, 0.15% was used as the dd-cfDNA%-only cutoff.

### 2.3. Biopsy-defined rejection

Biopsies were reviewed by local pathologists and were graded according to the International Society for Heart and Lung Transplantation classification schemes. ACR was graded according to the International Society for Heart and Lung Transplantation 2005 revised guidelines,^32^ whereas AMR was graded according to the 2013 pathologic criteria.^33^ AR included pAMR1 (H+), pAMR1 (I+), pAMR2 and pAMR3, ACR grade 2R and grade 3R, or a combination thereof. Nonrejection (non-AR) was defined as pAMR0 and ACR grades 0 or 1R. Histopathologic interpretation and grading were performed by local center pathologists.

### 2.4. Donor-specific antibodies

Donor-specific antibodies (DSAs) were measured locally at each site using the Luminex xMAP platform (Thermo Fisher Scientific). Cutoffs of 2000 and 3000 median florescence intensities were for diagnosis of a positive DSA test at University of Utah and University of California, San Diego, respectively.^34^

### 2.5. Statistical analysis

Descriptive statistics were reported using mean and standard deviation or median and interquartile range (IQR) for continuous variables. Range, consisting of the maximum and minimum values, was reported for other variables. Discrete variables were reported as numbers and percentages. The Mann-Whitney U test was used to assess the statistical significance between different groups. Two-tailed *P* values < .05 were considered statistically significant. Corrections for multiple comparisons were performed using the Benjamini-Hochberg method.

To account for repeated samples at different timepoints from the same patient, a generalized mixed effects regression model was used when comparing dd-cfDNA%, DQS, and total cfDNA in AR and non-AR samples. Rejection prevalence and performance metric calculations (sensitivity, specificity, negative predictive value [NPV], positive predictive value [PPV], and AUC) were estimated by bootstrap after running 10,000 iterations. In each iteration, only 1 sample per patient was randomly selected. The rejection prevalence of this cohort was used to calculate NPV and PPV.

Trends of cfDNA measures (total cfDNA, dd-cfDNA%, and DQS) over time since transplant were assessed using locally weighted scatterplot smoothing regression that estimated a weighted quadratic least square regression over a span of time.^35^ Performance of dd-cfDNA test algorithms was assessed by constructing receiver operating characteristic (ROC) curves and calculating the AUC and accuracy measures (eg, sensitivity and specificity). The AUC for the 2TA was estimated using a single-threshold function of the form (dd-cfDNA% / C_1_)^n^ + (DQS / C_2_)^n^, where C_1_ and C_2_ are the 2 thresholds. For sufficiently large values of n (n > 20), the output of the aforementioned single-threshold function matched the output of the 2TA in terms of risk categorization, and the single-threshold function allowed the estimation of the AUC using conventional methods. The contribution of DQS to dd-cfDNA% was estimated by the net reclassification index, which evaluates the proportion of samples that moved into different accurate and inaccurate categories. Spearman’s rank correlation coefficient ρ was calculated for correlation analysis. All statistical analyses were performed using Python 3.8.2.

## 3. Results

### 3.1. Characteristics of study population

A total of 808 dd-cfDNA samples from 187 HTx patients were eligible for the study (Fig. 1B). The median patient age at transplant was 57 years (IQR, 45–64), 75.9% (142/187) were male, 49.2% (92/187) were White, and patient median body mass index was 27.2 kg/m^2^ (IQR, 23.8–32.0 kg/m^2^; Table 1). The most common indications for HTx were nonischemic cardiomyopathy (63.6%) and ischemic cardiomyopathy (27.3%), and 7 patients (3.7%) had a prior HTx. At the time of transplant, 31.6% of patients had ventricular assist devices. The median time from transplant to first dd-cfDNA test draw was 69 days (IQR, 39–603 days) and patients had an average of 4 (range, 1–14) dd-cfDNA tests during the study. Across all EMBs included in the study, 9.2% (74/808) were for cause and the remainder were for surveillance indication. Patient demographics in a contemporaneous cohort of HTx recipients from the Scientific Registry of Transplant Recipients were similar to the study cohort (Supplementary Table S1).

### 3.2. dd-cfDNA fraction and dd-cfDNA quantity estimates

**Figure 2.**
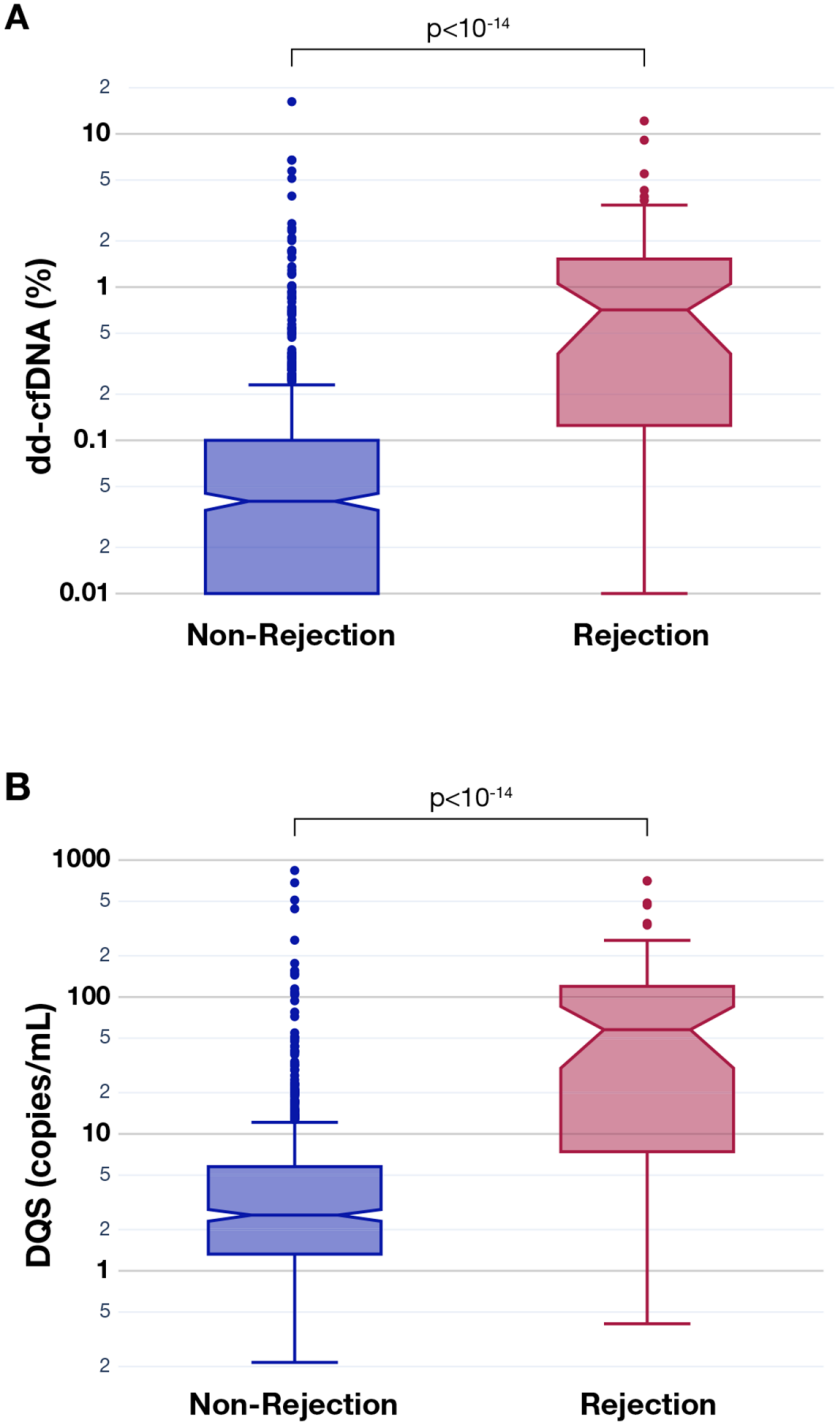
dd-cfDNA% and DQS in AR and non-AR samples. (A) dd-cfDNA% (%) and (B) DQS (copies/mL) in rejection vs non-rejection samples. Acute rejection samples (biopsy-proven) showed significantly increased dd-cfDNA% and DQS compared to nonrejection samples. The horizontal notched line represents the median value, and the top and bottom of each box show upper and lower limit of the IQR, and the whiskers represent the range. AR, acute rejection; dd-cfDNA, donor-derived cell-free DNA; DQS, donor-quantity score; interquartile range.

AR was detected in 41 of 808 (5.1%) biopsy-matched samples from 30 patients (Fig. 1B). Both the median dd-cfDNA% and median DQS were significantly higher in the AR group (dd-cfDNA % = 0.71% and DQS = 58 cp/mL) than in the non-AR group (dd-cfDNA% = 0.04% and DQS = 3 cp/mL; *P* < 10^−14^ for both comparisons; Fig. 2). The distribution of dd-cfDNA%, DQS, and total cfDNA for the non-AR, AR, and combined cohorts are shown in Supplementary Figure S1. The intra- and interpatient variance of total cfDNA levels was 15.2% and 36.4%, respectively. Fold change between the median and largest total cfDNA level within the same patient went up to a maximum of 15.7×, with 50th and 75th percentiles of 1.38 and 1.86, respectively. The maximum intrapatient fold change (ie, change from minimum to maximum) in total cfDNA was 54.0×. Linear mixed effect models of random pairwise comparisons of samples showed significant differences between AR and non-AR for both dd-cfDNA% and DQS (*P* =.034 and .001, respectively). No significant differences in median total cfDNA levels between the AR and non-AR groups were observed (6140 cp/mL vs 6970 cp/mL, *P* = .562; Supplementary Fig. S2).

**Figure 3.**
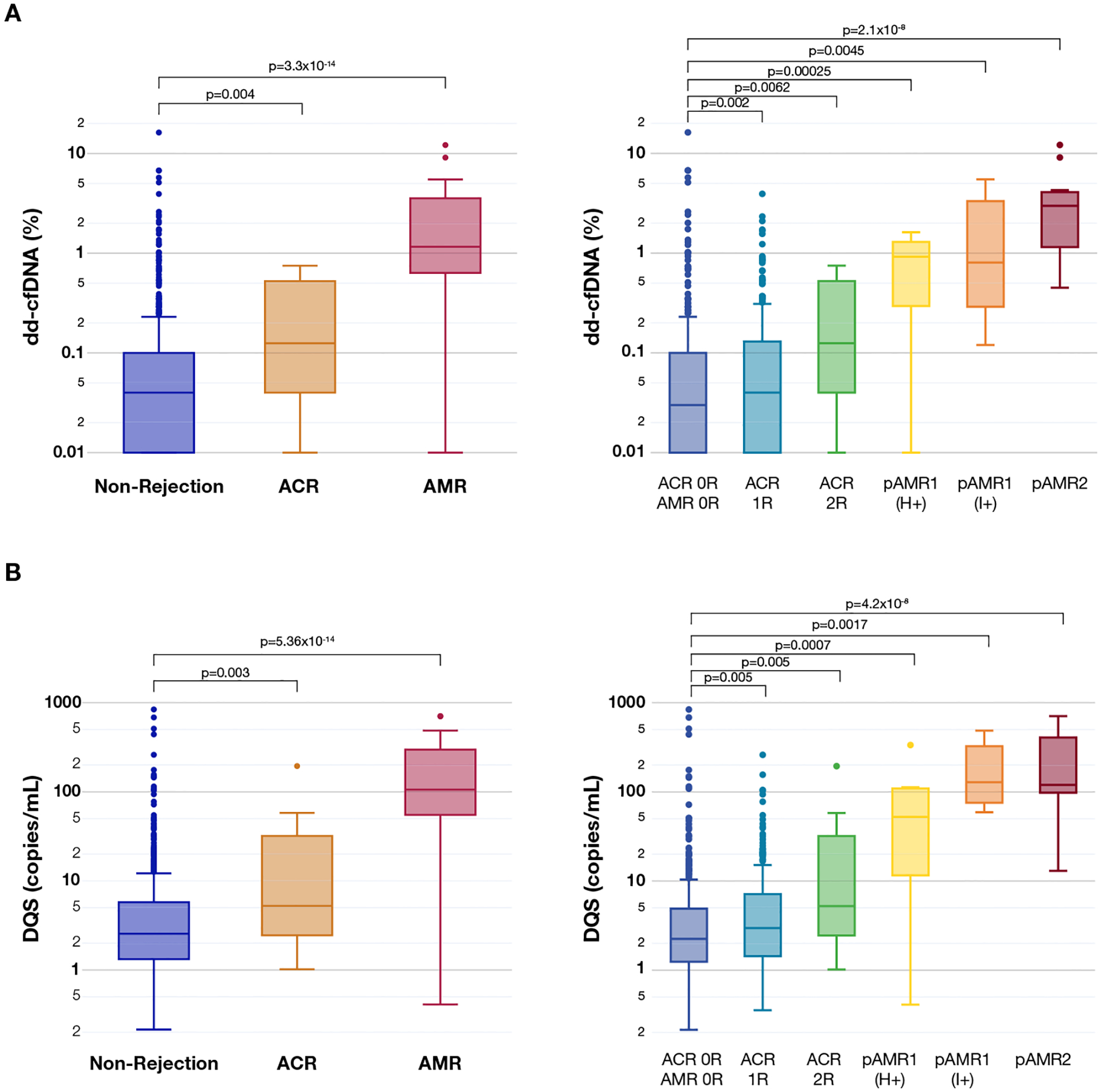
dd-cfDNA levels in patients stratified by biopsy diagnosis. (A) Both ACR and AMR show significantly increased dd-cfDNA% compared to nonrejection samples (left). ACR 1R and 2R show significantly increased dd-cfDNA% compared to non-rejection samples (right). AMR shows a trend for further increase in dd-cfDNA% compared to ACR. (B) Both ACR and AMR show significantly increased DQS compared to nonrejection samples. DQS shows a similar increase in values compared to dd-cfDNA% across different acute rejection grades (right). ACR, acute cellular rejection; AMR, antibody-mediated rejection; dd-cfDNA, donor-derived cell-free DNA; DQS, donor-quantity score.

The median dd-cfDNA% and DQS were significantly higher for both AMR (dd-cfDNA% = 1.16%; DQS = 106 cp/mL; *P* <.001 for both) and ACR (dd-cfDNA% = 0.13; DQS = 5 cp/mL; *P* =.004 for both) than for non-AR (dd-cfDNA% = 0.04, DQS = 3 cp/mL) (Fig. 3A, B).

### 3.3. Implementing a 2TA for the detection of AR

**Figure 4.**
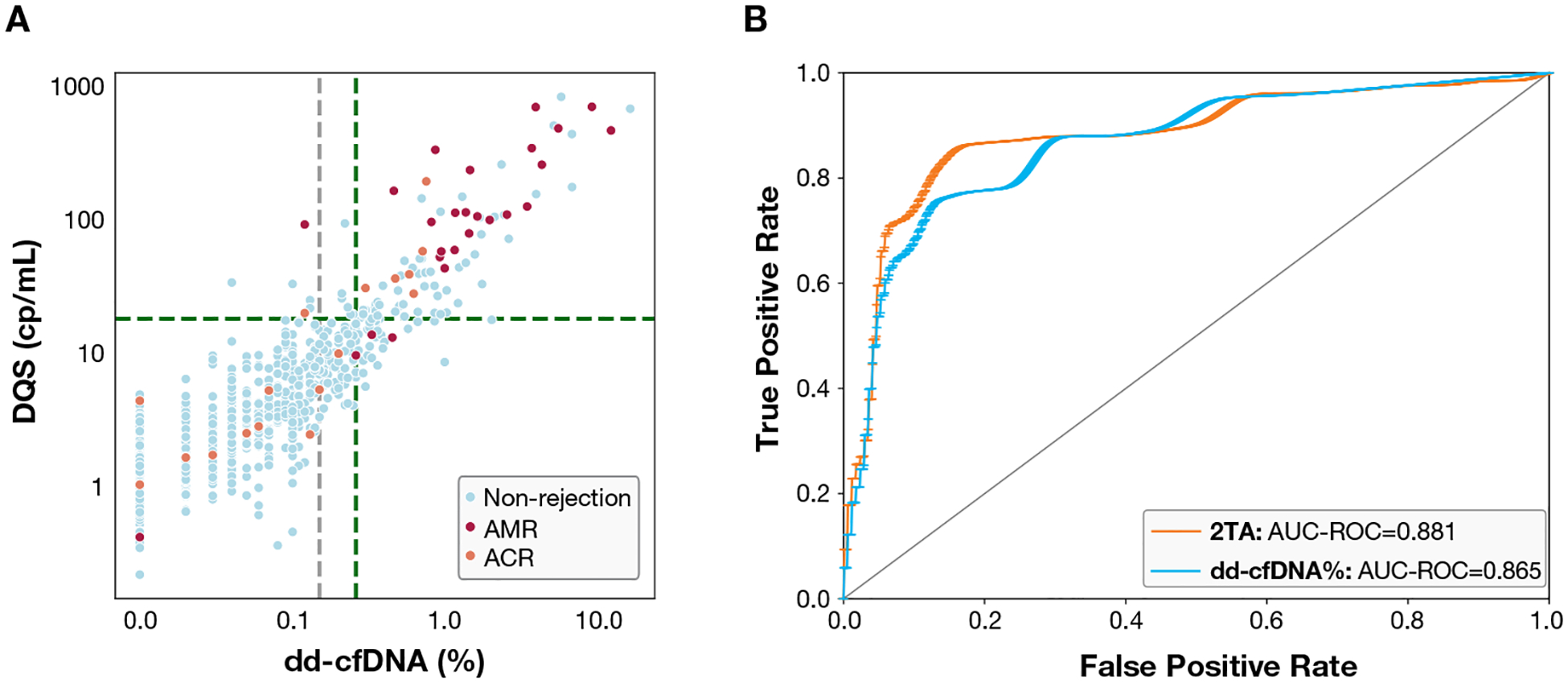
Performance of a 2-threshold algorithm. (A) Scatterplot of dd-cfDNA test samples; grey vertical line indicates the 0.15% threshold; green vertical line indicates 2TA threshold of 0.26%; green horizontal line indicates 2TA threshold of 18 cp/mL. (B) ROC of dd-cfDNA (%) and 2TA, AUC with bootstrapping. 2TA demonstrated improved testing performance compared to dd-cfDNA% alone. 2TA, 2-threshold algorithm; ACR, acute cellular rejection; AMR, antibody-mediated rejection; AUC, area under the curve; dd-cfDNA, donor-derived cell-free DNA; ROC, receiver operating characteristic.

The grid search identified 3 local maxima for the sum of sensitivity and specificity (Supplementary Table S2). Cutoff values of 0.26% and 18 cp/mL, for which exceeding either of these thresholds is interpreted as a positive result, were identified as values that best balanced sensitivity and specificity. Furthermore, selection of this combination ensured adequate identification of both ACR and AMR while still minimizing false positives. The 2TA yielded a sensitivity of 86.5% (95% CI, 70.4%−99.6%) and specificity of 83.6% (95% CI, 78.0%−89.1%), with NPV and PPV of 98.5% (95% CI, 96.7%−99.9%) and 33.58% (95% CI, 24.89%−42.26%), respectively (Fig. 4A). When compared to the use of the previously recommended dd-cfDNA% threshold of 0.15%, the 2TA showed improved performance (Table 2). The 2TA showed an AUC of 0.881 (95% CI, 0.782–0.979), compared with an AUC of 0.865 for dd-cfDNA% alone (95% CI, 0.767–0.962; *P* =.81; Fig. 4B).

When compared to dd-cfDNA% alone, the 2TA decreased the number of false positive findings by 37.3% (from 126 to 79) and increased the number of true negative findings (from 641 to 688), while not affecting the number of true positive or false negative findings (30 and 11, respectively) (Supplementary Fig. S3C). The 2TA resulted in a mean net reclassification index of 16.4% (standard deviation: 4.0%; *P* =.015). Of the 79 samples that were considered positive by the 2TA but did not have evidence of AR by histopathology, 74 had some other clinical feature known to be associated with elevated dd-cfDNA including a history of rejection, cardiac allograft vasculopathy (CAV), graft dysfunction, ACR-1R diagnosis, infection, and DSA positivity.

### 3.4. cfDNA trajectory over time

**Figure 5.**
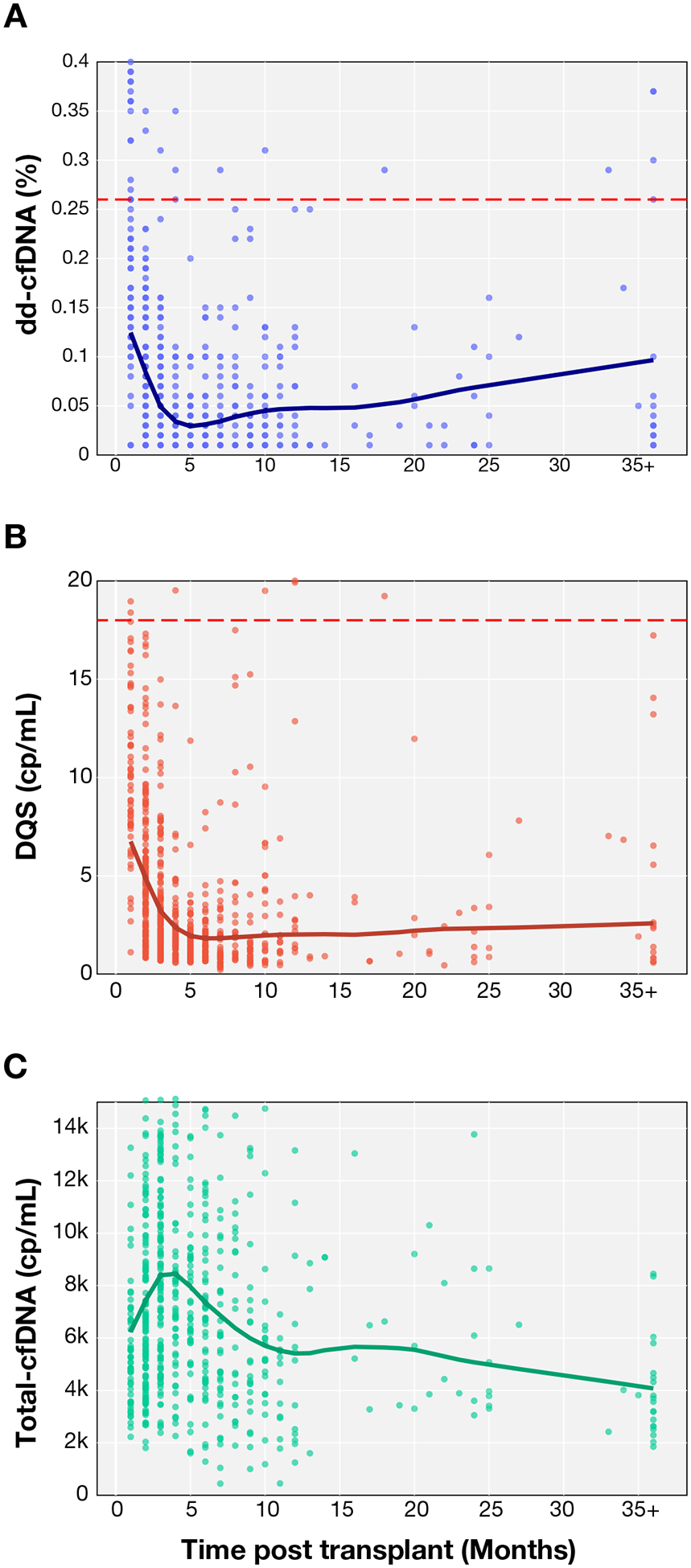
LOWESS smoothed trajectories of (A) dd-cfDNA%, (B) DQS, (C) total cfDNA over time. dd-cfDNA% (A) shows an increasing trend over time after 7 months posttransplant, DQS (B) remains relatively unchanged, and total cfDNA (C) shows a decreasing trend after 7 months posttransplant. dd-cfDNA, donor-derived cell-free DNA; DQS, donor-quantity score; LOWESS, locally weighted scatterplot smoothing.

We assessed the differences in dd-cfDNA%, DQS, and total cfDNA trends over time posttransplant. The locally weighted scatterplot smoothing regression modeling of 145 stable patients (defined as those without AR or CAV) over 36 months posttransplant showed the trends in dd-cfDNA% (Fig. 5A), DQS (Fig. 5B), and total cfDNA (Fig. 5C).

### 3.5. Associations of dd-cfDNA and other clinical factors

Among the 808 EMBs included in the study, median dd-cfDNA % and DQS were significantly higher among patients with a for-cause biopsy (0.22% [0.06–1.09%]; 11 cp/mL [3–99 cp/mL]; n = 74) than for a surveillance biopsy (0.04% [0.01–0.11%], 3 cp/mL [1–6 cp/mL]; n = 734; *P* <.0001). Median dd-cfDNA% and DQS were also significantly higher among DSA-positive cases (0.22% [0.08–1.00%]; 8 cp/mL (2–94 cp/mL) than among DSA-negative cases (0.04% [0.01–0.11%]; 3 cp/mL (1–7 cp/mL); *P* < .0001; Supplementary Fig. S4). We also assessed the relationship of dd-cfDNA with graft dysfunction. Among 659 samples with contemporaneous left ventricular ejection fraction (LVEF) reported, both dd-cfDNA% and DQS were significantly higher in samples with LVEF <40% than in those with LVEF ≥40% (dd-cfDNA%: 0.58% [0.145–1.45%] vs 0.04% [0.01–0.11%]; DQS: 75 [19–162] vs. 2 [1–14] cp/mL; *P* < 10^−6^ for both comparisons). These differences remained significant when we considered the AR and non-AR cohorts separately (Supplementary Fig. S5). Furthermore, across patients with non-AR, both dd-cfDNA% and DQS were significantly higher in those that were diagnosed with CAV than in those that did not have evidence of CAV (dd-cfDNA %: 0.13% (0.04–0.245%) vs. 0.04% (0.01–0.11%), *P* =.006; DQS: 4.2 (2.2–13.2) vs 2.2 (1.12–7.10) cp/mL; *P* =.04) (Supplementary Fig. S6).

## 4. Discussion

In this study, we applied novel analytical methods to consider DQS along with dd-cfDNA% after HTx. We show that a 2TA that combines dd-cfDNA% with DQS thresholds improved accuracy for AR detection compared with dd-cfDNA% alone.

The commonly used approach of assessing dd-cfDNA as a fraction of total cfDNA^15,18,20,36^ can be influenced by changes in recipient derived-cfDNA (rd-cfDNA), which makes up the majority of circulating total cfDNA. Studies have shown that levels of rd-cfDNA fluctuate in both kidney and HTx patients,^22,28,29,37^ which can, in turn, significantly alter the reported dd-cfDNA%, even in the absence of changes in DQS.

In our prior study of dd-cfDNA% in adult HTx patients, we found 0.15% to be an optimal threshold for discriminating AR from non-AR.^19^ In the present study, we combined dd-cfDNA% with DQS into the 2TA approach, incorporating a higher dd-cfDNA% cutoff than previously established, such that either metric above its respective threshold is classified as positive. Our results indicate that this approach may improve the performance of dd-cfDNA testing by reducing false positive results, without reduction in sensitivity (Fig. 4A). If implemented in clinical practice, this could potentially further reduce the number of EMBs indicated compared to using dd-cfDNA% alone.

Our findings corroborate those of Oellerich et al.,^28^ who compared the performance characteristics of dd-cfDNA% to the absolute quantity of dd-cfDNA, as determined by digital droplet PCR, in kidney transplant. The authors found an improvement in AUC (0.83 vs 0.73), sensitivity (73% vs 68%) and specificity (73% vs 68%) when using absolute quantity of dd-cfDNA to detect rejection compared to dd-cfDNA%.

Here, the inclusion of DQS was useful in identifying AR cases in which the dd-cfDNA% was low due to the variability of total cfDNA levels. An example of such a case is a 64-year-old HTx recipient in our study who had biopsy-proven ACR 2R rejection 572 days posttransplant. Concurrent dd-cfDNA testing yielded a dd-cfDNA% of 0.12% and DQS of 19.8 cp/mL. The patient’s total cfDNA was significantly elevated (16500 cp/mL; ~2.5× the median), potentially from concomitant infection with *Clostridium difficile*, methicillin-sensitive *Staphylococcus aureus*, and septic arthritis. The increase in total cfDNA decreased the dd-cfDNA% to less than the 0.15% cutoff. However, the patient’s result was positive using the 2TA because DQS was greater than the cutoff of 18 cp/mL. Similar cases have also been described by others. In a case series report by Oellerich et al,^37^ the authors propose explanatory clinical variables for the discrepancies between dd-cfDNA% and dd-cfDNA quantification. They present 5 patients who had normal dd-cfDNA% but increased absolute dd-cfDNA levels. Four of these patients exhibited clinical signs of allograft injury or biopsy-proven rejection, suggesting that relying solely on dd-cfDNA% may lead to false negatives. Three of the 4 patients had viral or bacterial infections likely causing an increase in total cfDNA levels. In the BIODRAFT study (NCT03477383), Böhmer et al.^29^ compared absolute dd-cfDNA quantification to dd-cfDNA% in 52 HTx patients and found that both metrics were increased during biopsy-proven rejection. However, they noted high intra- and interpatient variability in total cfDNA levels due to factors such as infection and bleeding.^38^ In one instance, infection caused a 5-fold increase in rd-cfDNA, suppressing dd-cfDNA% despite AR (ACR grade 2R). However, the quantity of cfDNA was markedly elevated. Similarly, we observed significant variability in total cfDNA, with one subject showing a 54-fold change. In the present study, we found that dd-cfDNA% gradually increased in stable patients, although still remaining below thresholds, beginning 7 months after HTx, potentially due to changes in total cfDNA. However, DQS remained reasonably constant.

A complicating factor in this analysis is that dd-cfDNA can sometimes be associated with future rejection or other non-rejection events, which can result in false positive results. Examples include cytomegalovirus infection, graft dysfunction, and CAV. Additional insights into the dd-cfDNA results determined to be false positive and false negative as related to a concurrent EMB were provided by De Vlaminck et al.^16^ The authors reported that many patients with false positive results later developed biopsy-proven rejection, whereas many patients with false negative results had good allograft function in follow-up. More recently, Agbor-Enoh et al.^15^ found that dd-cfDNA% started to increase at a median of 3.2 months before AMR diagnosis, suggesting that samples drawn during this window could appear to be false positives. Of the 79 samples that were considered false positive in our analysis, 74 had other clinical findings known to be associated with increased dd-cfDNA.

The approach and results of our study are also aligned with the findings of Halloran et al,^31^ who reported that in the Trifecta kidney transplant study, the combination of dd-cfDNA fraction and quantity was more powerful than either dd-cfDNA fraction or quantity alone, and the authors validated a novel 2TA incorporating both variables in kidney transplant.

Our study has limitations, including a cohort limited to HTx patients from 2 programs in the United States, and thus may not necessarily represent the experience of other centers with different patient demographics and variations in post-HTx management. The cross-sectional study design in which patients were primarily selected at the time of biopsy introduces a possibility of selection bias and prevents analyses requiring longitudinal dd-cfDNA results, such as assessing the value of dd-cfDNA in monitoring treatment response. The determination of the thresholds of the 2TA was optimized for this study cohort, and validation of these thresholds in a separate population is warranted. Consideration of DQS as an independent biomarker for AR could have been an alternate approach in our study. We opted for the 2TA approach, as both DQS and dd-cfDNA% have independent value, with each metric better poised to capture rejection in individuals with total cfDNA that is either particularly high or low, respectively. Adding a DQS threshold allowed us to increase the dd-cfDNA% threshold, reducing the false positive rate considerably without affecting the false negative rate. We therefore opted for combining DQS threshold with dd-cfDNA%, an approach that has also been used in kidney transplant.^30,31^ An ongoing multicenter study of dd-cfDNA will allow us to externally validate the findings of this study in a diverse cohort of heart transplant patients (Trifecta--Heart NCT04707872). Finally, the reference standard used in our study—the histopathologic interpretation of EMB samples used to define rejection—has well established limitations that need to be considered when interpreting the study results.^39^

In conclusion, the low diagnostic yield and the risks of EMB have resulted in an increased use of noninvasive graft surveillance after HTx, namely dd-cfDNA%. We report that combining dd-cfDNA% with DQS may improve the accuracy of dd-cfDNA testing for AR. If our findings can be validated in additional patient cohorts, this approach may result in further reduction in the use of surveillance EMBs.

## Supplementary Material

Suppmaterial

## Figures and Tables

**Table 1:** Baseline characteristics.

Characteristic	All patients (N = 187)	Patients with rejection (n = 30)	Patients with no rejection (n = 157)	*P*
*Recipient characteristics*				
Age (y), median (IQR)	57.0 (45.0–64.0)	52.5 (36.0–63.0)	59.0 (46.0–64.0)	.1343
Sex, n (%)				
Male	142 (75.9%)	23 (76.7%)	119 (75.8%)	1.0000
Female	45 (24.1%)	7 (23.3%)	38 (24.2%)	
BMI (kg/m^2^), median (IQR)	27.2 (23.8–32.0)	28.2 (23.5–31.5)	27.1 (23.8–32.0)	.8180
Race/ethnicity, n (%)				
White	92 (49.2%)	13 (43.3%)	79 (50.3%)	.4490
Hispanic	41 (21.9%)	8 (26.7%)	33 (21.0%)	
African American	24 (12.8%)	5 (16.7%)	19 (12.1%)	
Asian	19 (10.2%)	1 (3.3%)	18 (11.5%)	
Other	11 (5.9%)	3 (10.0%)	8 (5.1%)	
Indication for transplant, n (%)				
NICM	119 (63.6%)	21 (70.0%)	98 (62.4%)	.6052
ICM	51 (27.3%)	6 (20.0%)	45 (28.7%)	
Congenital	8 (4.3%)	1 (3.3%)	7 (4.5%)	
Retransplantation	7 (3.7%)	1 (3.3%)	6 (3.8%)	
Other/missing	2 (1.1%)	1 (3.3%)	1 (0.6%)	
LVAD at HTx, n (%)	59 (31.6%)	10 (33.3%)	49 (31.2%)	.9881
Sensitization (PRA ≥10%), n (%)	21 (11.2%)	3 (10.0%)	18 (11.5%)	1.0000
Donor-specific antibodies at the time of biopsy, n (%)				
Yes	22 (11.8%)	11 (36.7%)	11 (7.0%)	<.0001
No	141 (75.4%)	13 (43.3%)	128 (81.5%)	
Not Assessed	24 (12.8%)	6 (20.0%)	18 (11.5%)	
LVEF (%), median (IQR)	62.0 (58.0–67.0)	61.5 (59.0–66.0)	62.0 (58.0–68.0)	.2482
Recipient CMV, n (%)				
Positive	123 (65.8%)	21 (70.0%)	102 (65.0%)	.7658
Negative	57 (30.5%)	8 (26.7%)	49 (31.2%)	
Not available	7 (3.7%)	1 (3.3%)	6 (3.8%)	
CMV recipient/donor match/mismatch, n (%)				
R+/D+	74 (39.6%)	14 (46.7%)	60 (38.2%)	.6238
R−/D−	20 (10.7%)	4 (13.3%)	16 (10.2%)	
R+/D−	46 (24.6%)	6 (20.0%)	40 (25.5%)	
R−/D+	37 (19.8%)	4 (13.3%)	33 (21.0%)	
R_NA_/D_NA_	7 (3.7%)	1 (3.3%)	6 (3.8%)	
R or D NA	3 (1.6%)	1 (3.3%)	2 (1.3%)	
*dd-cfDNA sample characteristics*				
No. of dd-cfDNA samples/patient				
Mean (range)	4.0 (1.0–14.0)	3.5 (1.0–14.0)	4.0 (1.0–11.0)	.3740
Time from transplant to first dd-cfDNA sample (d)				
Median (IQR)	69.0 (39.0–603.0)	571.5 (50.8–1302.8)	51.0 (39.0–383.0)	.0011
Time from transplant to last dd-cfDNA sample (d)				
Median (IQR)	292.0 (178.5–725.5)	715.5 (348.5–1302.8)	248.0 (172.0–564.0)	<.0001
Biopsy characteristics	All samples (N = 808)	Samples with rejection (n = 41)	Samples with no rejection (n = 767)	P
Biopsy type, n (%)				<.0001
For cause	74 (9.2%)	20 (48.8%)	54 (7.0%)	
Per protocol	734 (90.8%)	21 (51.2%)	713 (93.0%)	
Rejection type and grade; n (%)				
ACR	16	16	NA	NA
ACR2R	16 (100.0%)	16 (100.0%)		
ACR3R	0 (0.0%)	0 (0.0%)		
AMR	25	25		
pAMR1 (H+)	9 (36.0%)	9 (36.0%)		
pAMR1 (I+)	4 (16.0%)	4 (16.0%)		
pAMR2	12 (48.0%)	12 (48.0%)		

*P* values assess the significance of differences in metrics between patients with rejection and those with no rejection.

ACR, acute cellular rejection; AMR, antibody-mediated rejection; BMI, body mass index; CMV, cytomegalovirus; dd-cfDNA, donor-derived cell-free DNA; HTx, heart transplantation; ICM, ischemic cardiomyopathy; IQR, interquartile range; LVAD, left ventricular assist device; LVEF, left ventricular ejection fraction; NA, not applicable; NICM, nonischemic cardiomyopathy; PRA, panel reactive antibody.

**Table 2:** Performance characteristics of dd-cfDNA% and of 2TA in HTx patients^a^.

Characteristic	dd-cfDNA% (cutoff: 0.15%)	2TA (cutoffs: 0.26% or 18 cp/mL)
Sensitivity (AR)	78.20% (58.38%, 97.32%)	86.48% (70.35%, 99.57%)
Specificity	76.92% (70.61%, 83.24%)	83.57% (78.01%, 89.12%)
Positive predictive value^b^	25.16% (18.09%, 32.22%)	33.58% (24.89%, 42.26%)
Negative predictive value^b^	97.28% (94.85%, 99.63%)	98.48% (96.68%, 99.95%)
AUC	0.865 (0.767, 0.962)	0.881 (0.782, 0.979)
LR+	3.41 (2.34, 4.96)	5.30 (3.59, 7.83)
LR−	0.28 (0.12, 0.72)	0.16 (0.05, 0.57)

AR, acute rejection; AUC, area under the curve; dd-cfDNA, donor-derived cell-free DNA; HTx, heart transplantation; LR, likelihood ratio; 2TA, 2-threshold algorithm.

a Data are presented as mean (95% confidence interval).

b Based on a cohort rejection prevalance of 8.74%.

## Data Availability

The data are not publicly available. Researchers can reach out to the corresponding author with a request to review deidentified data.

## Footnotes

1 Prospera is a Natera-owned trademark

## Abbreviations:

ACR: acute cellular rejection
AMR: antibody-mediated rejection
AR: acute rejection
AUC: area under the curve
CAV: cardiac allograft vasculopathy
CI: confidence interval
cp: genomic copies
dd-cfDNA: donor-derived cell-free DNA
DSA: donor-specific antibody
DQS: donor-quantity score
EMB: endomyocardial biopsy
HTx: heart transplantation
IQR: interquartile range
LVEF: left ventricular ejection fraction
NPV: negative predictive value
PPV: positive predictive value
PCR: polymerase chain reaction
rd-cfDNA: recipient-derived cfDNA
ROC: receiver operating characteristic
2TA: 2-threshold algorithm
