# Supplementary material for: A two-threshold algorithm using donor-derived cell-free DNA fraction and quantity to detect acute rejection after heart transplantation
Source: Am J Transplant. Author manuscript; Available in PMC 2025 Sep 15. (PMC12435761; doi:10.1016/j.ajt.2025.04.021)
Supplement: Suppmaterial [file NIHMS2109705-supplement-Suppmaterial.pdf]

SUPPLEMENTARY MATERIAL

Kim, PJ, et al. “A Two-Threshold Algorithm using Donor-derived Cell-free DNA Fraction and Quantity to Detect Acute Rejection After Heart Transplantation”

Table S1: Comparison of summary statistics between contemporaneous SRTR cohort and study cohort

	**SRTR**	**Study Cohort**
Subjects	N=10298	N=187
**Age at transplantation:** Median [IQR]	56.0 [18.0]	57 [45-64]
Biological sex		
Female	2794 (27.1%)	45 (24.1%)
Male	7504 (72.9%)	142 (75.9%)
**BMI:** Median [IQR]	27.5 [7.40]	27.2 [23.9, 32.0]
Sensitization at transplantation (cPRA >= 10%)		
Yes	1866 (18.1%)	21 (11.2%)
No	8432 (81.9%)	
Race		
Asian	386 (3.7%)	19 (10.2%)
Black	2589 (25.1%)	24 (12.8%)
Multiracial	48 (0.5%)	
Native American	59 (0.6%)	
Pacific Islander	43 (0.4%)	
White	7082 (68.8%)	92 (49.2%)
Unreported	91 (0.9%)	
Ethnicity		
Hispanic/Latino	1229 (11.9%)	41 (21.9%)
Non-Hispanic/Non-Latino	9069 (88.1%)	
Primary diagnosis for transplantation		
ICM	2794 (27.1%)	51 (27.3%)
NICM	6555 (63.7%)	119 (63.6%)
Congenital disease	420 (4.1%)	8 (4.3%)
Recipient CMV Status		
Positive	5573 (54.1%)	123 (65.8%)
Negative	4208 (40.9%)	57 (30.5%)
Missing	517 (5.0%)	7 (3.7%)

SUPPLEMENTARY MATERIAL

Kim, PJ, et al. "A Two-Threshold Algorithm using Donor-derived Cell-free DNA Fraction and Quantity to Detect Acute Rejection After Heart Transplantation"

Table S2: Performance metrics for dd-cfDNA%/DQS combinations identified

	**dd-cfDNA%: 0.33%<br>DQS: 16 cp/mL**	**dd-cfDNA%: 0.26%<br>DQS: 18 cp/mL**	**dd-cfDNA%: 0.20%<br>DQS: 18 cp/mL**
**Sensitivity**	85.30%<br>(68.60%, 99.32%)	86.48%<br>(70.35%, 99.57%)	86.95%<br>(71.05%, 99.66%)
**Specificity**	86.43%<br>(81.30%, 91.56%)	83.57%<br>(78.01%, 89.12%)	80.44%<br>(74.50%, 86.39%)
**PPV**	37.67%<br>(27.58%, 47.75%)	33.58%<br>(24.89%, 42.26%)	29.93%<br>(22.43%, 37.44%)
**NPV**	98.40%<br>(96.61%, 99.92%)	98.48%<br>(96.68%, 99.95%)	98.47%<br>(96.64%, 99.96%)

SUPPLEMENTARY MATERIAL

Kim, PJ, et al. “A Two-Threshold Algorithm using Donor-derived Cell-free DNA Fraction and Quantity to Detect Acute Rejection After Heart Transplantation”

Figure S1: cfDNA Distribution among dd-cfDNA test samples

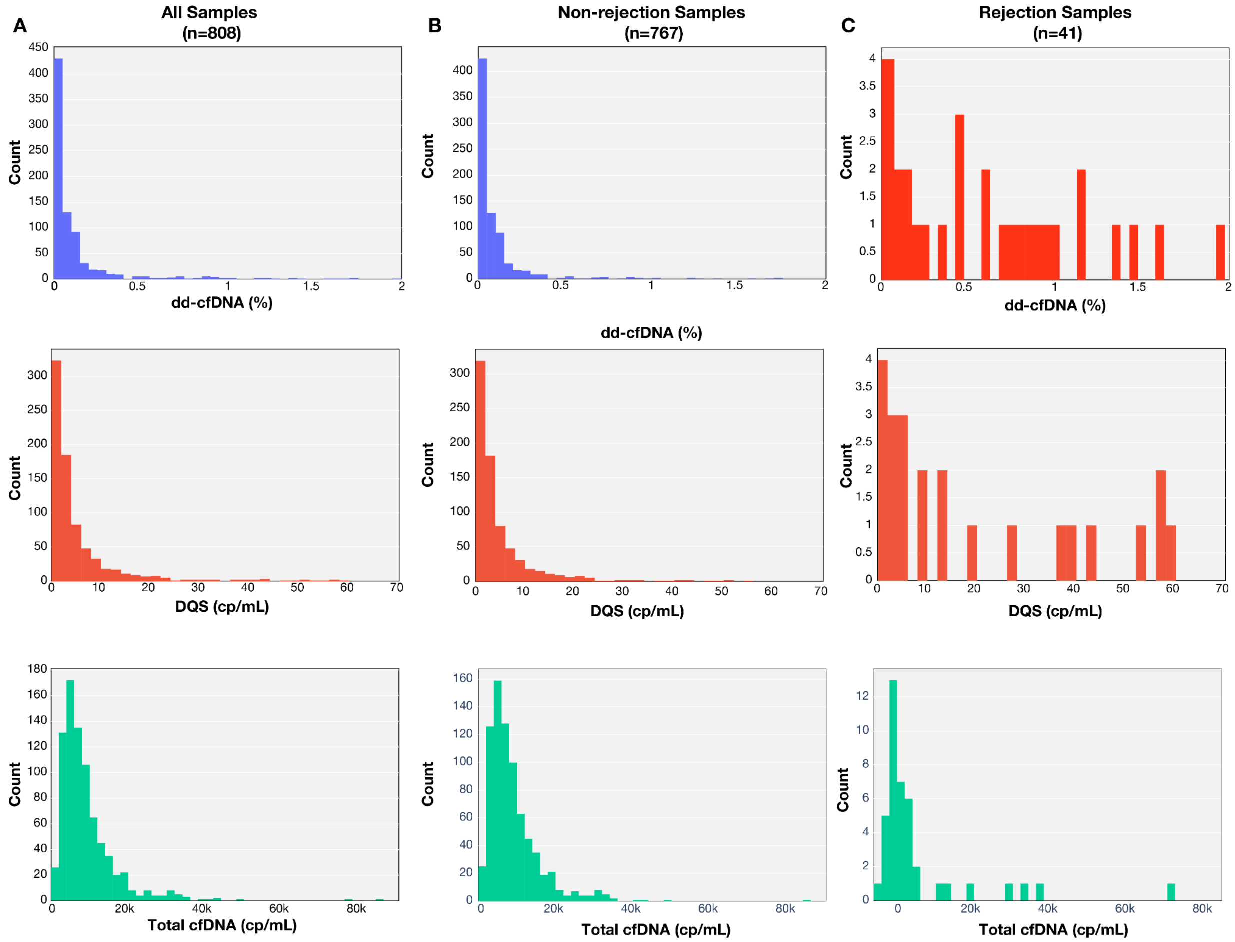

SUPPLEMENTARY MATERIAL

Kim, PJ, et al. "A Two-Threshold Algorithm using Donor-derived Cell-free DNA Fraction and Quantity to Detect Acute Rejection After Heart Transplantation"

Figure S2: No significant differences between total cfDNA levels in AR and non-rejection

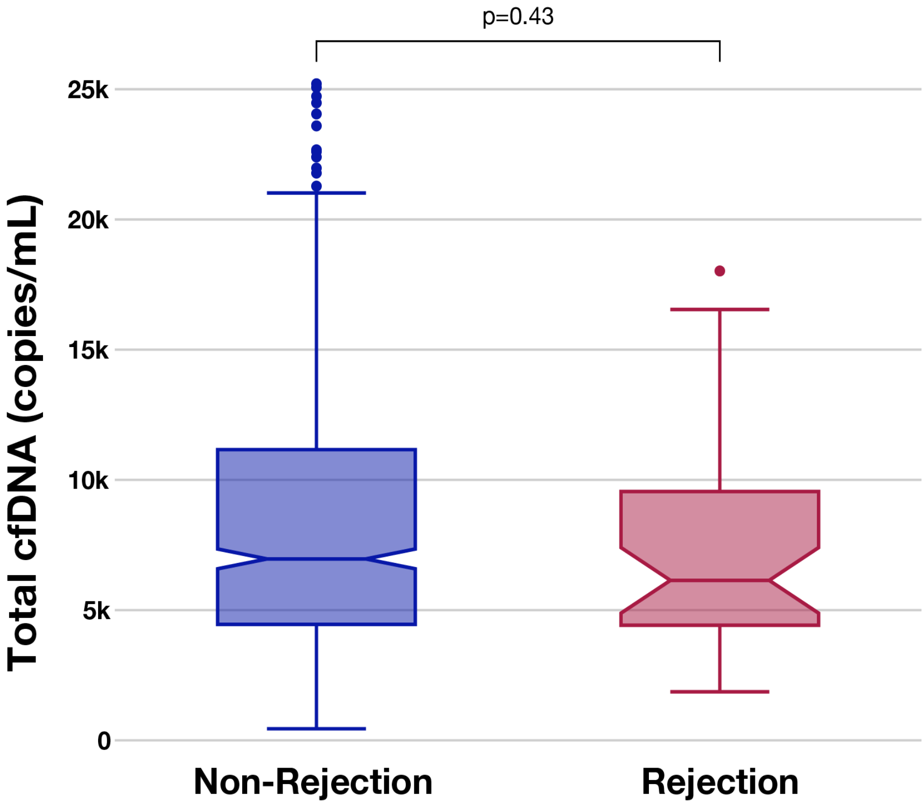

SUPPLEMENTARY MATERIAL

Kim, PJ, et al. "A Two-Threshold Algorithm using Donor-derived Cell-free DNA Fraction and Quantity to Detect Acute Rejection After Heart Transplantation"

Figure S3: Sample categorization using dd-cfDNA% vs. 2TA.

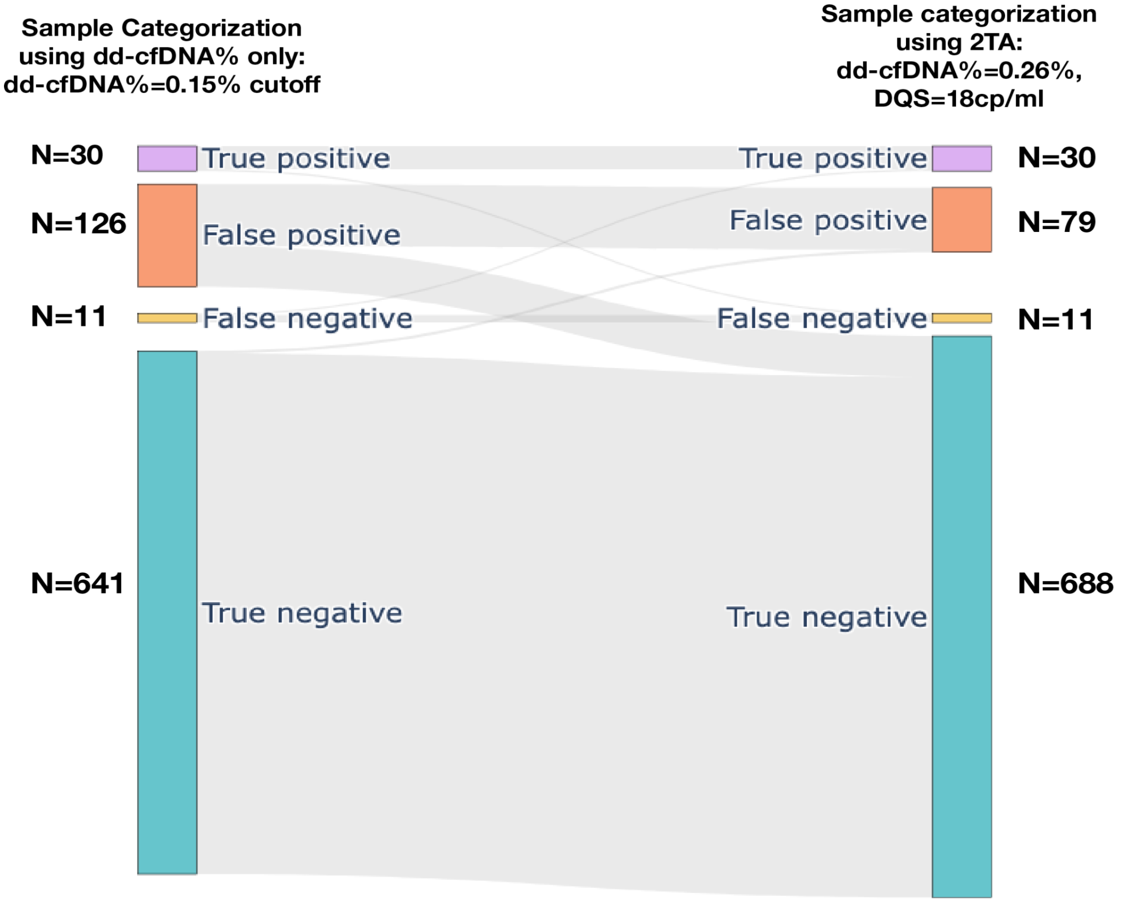

SUPPLEMENTARY MATERIAL

Kim, PJ, et al. "A Two-Threshold Algorithm using Donor-derived Cell-free DNA Fraction and Quantity to Detect Acute Rejection After Heart Transplantation"

Figure S4: Association between dd-cfDNA (A) fraction and (B) DQS with DSA.

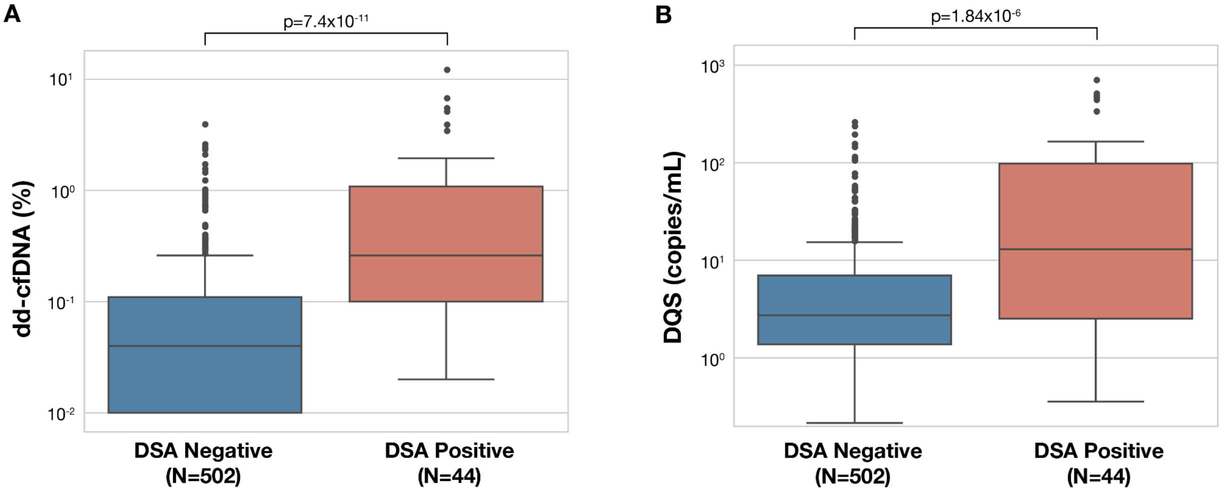

	DSA Negative (A)	DSA Positive (A)	DSA Negative (B)	DSA Positive (B)
**Median (IQR)**	0.04% (0.01 - 0.11)	0.22% (0.08 - 1.005)	2.7cp/mL (1.4 - 7.0)	8.5cp/mL (2.5 - 94.1)

SUPPLEMENTARY MATERIAL

Kim, PJ, et al. “A Two-Threshold Algorithm using Donor-derived Cell-free DNA Fraction and Quantity to Detect Acute Rejection After Heart Transplantation”

Figure S5: Association between dd-cfDNA and LVEF.

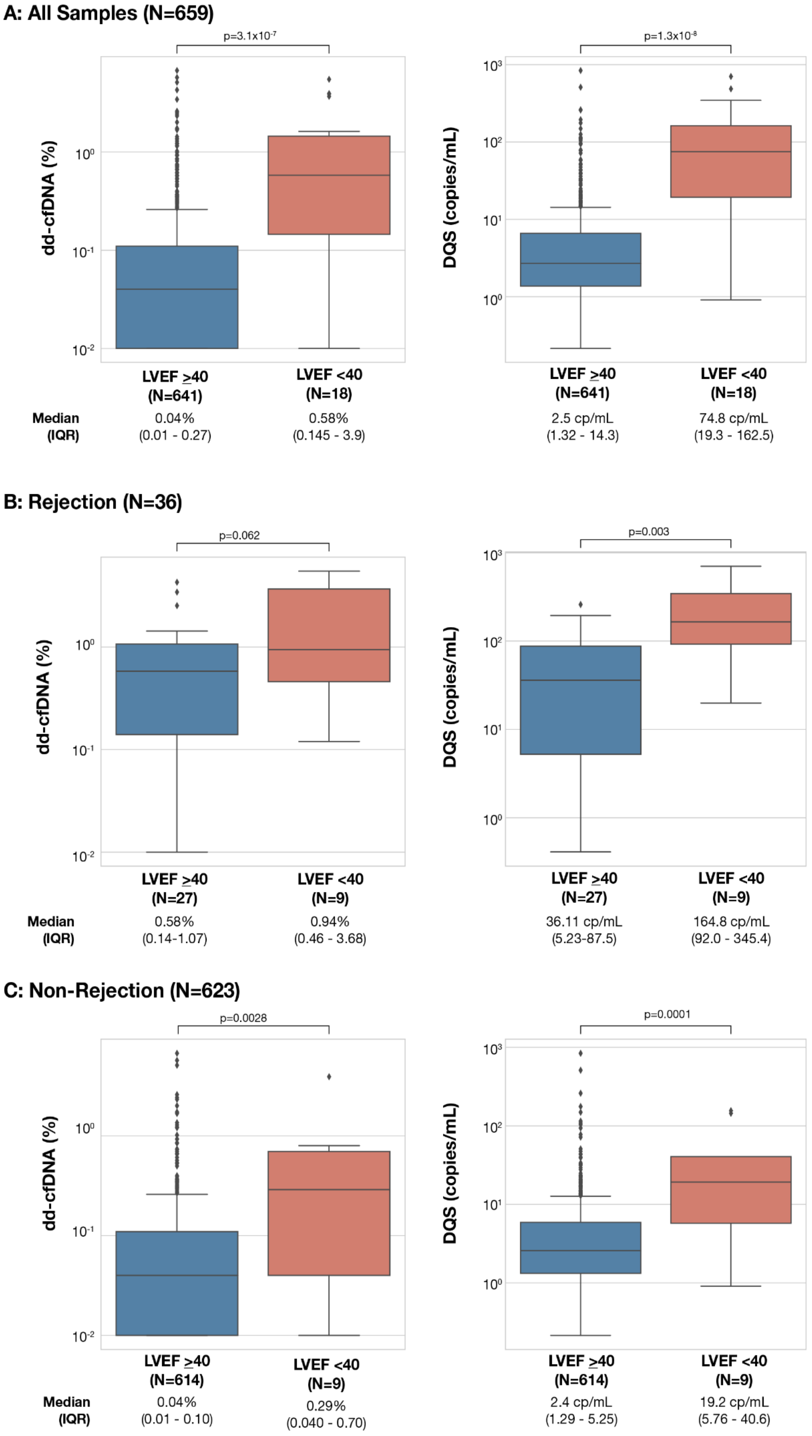

SUPPLEMENTARY MATERIAL

Kim, PJ, et al. “A Two-Threshold Algorithm using Donor-derived Cell-free DNA Fraction and Quantity to Detect Acute Rejection After Heart Transplantation”

Figure S6: Association between dd-cfDNA and CAV

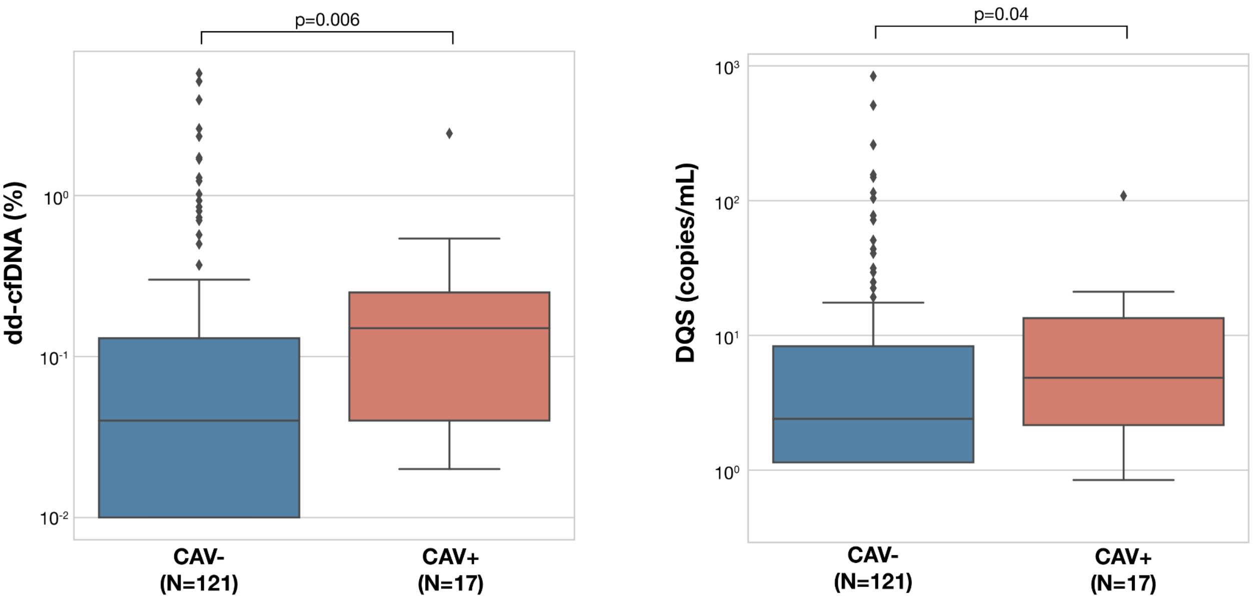

**Median (IQR)**	0.04% (0.01 - 0.11)	0.13% (0.04 - 0.245)	2.2 cp/mL (1.12 - 7.10)	4.2 cp/mL (2.2 - 13.2)
